# Hereditary Diffuse Gastric Cancer—Update Based on the Current Consort Recommendations

**DOI:** 10.3390/curroncol29040199

**Published:** 2022-03-30

**Authors:** Christoph Treese, Britta Siegmund, Severin Daum

**Affiliations:** Department for Medicine (Gastroenterology, Infectious Diseases, Rheumatology), Charité—Universitätsmedizin Berlin, Corporate Member of Freie Universität Berlin and Humboldt-Universität zu Berlin, 12203 Berlin, Germany; christoph.treese@charite.de (C.T.); britta.siegmund@charite.de (B.S.)

**Keywords:** hereditary diffuse gastric cancer, *CDH1* mutation, *CTNNA1* mutation, hereditary lobular breast cancer, hereditary diffuse gastric cancer-like, familial intestinal gastric cancer, prophylactic gastrectomy, screening endoscopy

## Abstract

Hereditary diffuse gastric cancer (HDGC) is an autosomal dominant inherited cancer syndrome that has been associated with a mutation of the *CDH1*, and rarely the *CTNNA1* gene, respectively. HDGC is characterized histologically by multifocal growth and signet ring cells in the gastric mucosa and lobular type breast cancer. In cases of a proven pathogenic *CDH1* mutation, a prophylactic gastrectomy, or alternatively, an annual surveillance gastroscopy in expert centers is recommended. Additionally, MR imaging of the breast should be performed annually starting from the age of 30, to detect lobular breast cancer. In 2020, the International Gastric Cancer Linkage Consortium (IGCLC) additionally defined new clinical groups with specific recommendations: (1) the group of patients with a proven mutation in the *CDH1* gene, but exclusive manifestation as lobular breast cancer, was defined as hereditary lobular breast cancer (HLBC); (2) the group, which clinically fulfills familial HDGC criteria, in the absence of a relevant mutation, was designated as HDGC-like. This update summarizes relevant aspects of hereditary gastric cancer and the current recommendation criteria of the IGCLC published in 2020.

## 1. Introduction

Globally, gastric cancer remains the fourth leading cause of tumor death despite the declining Helicobacter pylori infection rate [1]. Although a familial cluster has been observed in about 10% of patients affected by gastric cancer, the causes are largely unknown. Assignment to a hereditary syndrome has so far been successful in only 1–3% of patients, including those with hereditary diffuse gastric cancer (HDGC), familial intestinal gastric cancer (FIGC), and gastric adenocarcinoma with proximal gastric polyposis (gastric adenocarcinoma and proximal polyposis of the stomach (GAPSS) [2,3,4,5]. While no mutations have yet been identified for FIGC, HDGC was shown to be associated with a mutation in the *CDH1* gene [4]. After the familial cluster in Maori families in New Zealand was first described in 1964, the underlying *CDH1* gene was not detected until 1998 [6,7]. *CDH1* encodes the protein E-cadherin, a cell-cell adhesion molecule, which is responsible for epithelial integrity, binds to α1-catenin, and inhibits cell proliferation. A small proportion of patients have mutations in the *CTNNA1* gene, which encodes α1-catenin [8]. Pathognomonic for HDGC is the multifocal appearance of signet ring cell foci, which often escape endoscopic detection and may histologically appear as pathognomonic pagetoid spreading (see Table 1). An association with the occurrence of a cleft lip and palate has repeatedly been described [9].

Due to the increasing genetic diagnostics, the experience with this clinical picture has increased significantly. Above all, the testing for hereditary breast cancer, and specifically for the histological subtype of lobular breast cancer, has expanded the understanding of the disease, and led to the description of the entity “hereditary lobular breast cancer” (HLBC). The International Gastric Cancer Linkage Consortium (IGCLC), a group of experts from several disciplines that met for the first time in 1999, summarized the latest findings in 2020 and published them as expert recommendations [9,10]. These include, as essential innovations, the expansion of the screening criteria, the description of the above-mentioned HLBC entity (without the occurrence of gastric cancer in those affected) and the identification of the HDGC-like subgroup meeting the family screening criteria for HDGC, but with missing mutations in the *CDH1* and *CTNNA1* genes.

## 2. Definition and Epidemiology

### 2.1. Hereditary Diffuse Gastric Cancer (HDGC)

A population incidence of 5/100,000 is currently assumed for the HDGC. *CTNNA1* gene mutation (carried out in case of *CDH1* negativity) is correspondingly lower than 1/100,000 [11]. In individual population groups such as the Maori in New Zealand, the incidence is significantly higher, although more precise data are lacking. If the criteria for an HDGC according to the IGCLC are met, mutations in the *CDH1* or *CTNNA1* genes are found in 10 to 50% of cases [12]. The different hit rates are explained by the underlying incidences for gastric cancer: fewer mutations (approx. 20%) are found in high-incidence countries such as Portugal or Italy, which is in contrast to low-incidence countries such as the USA or Great Britain [13]. Based on the updated screening recommendations from 2020, an even lower positive rate of around 10% may be assumed for high-incidence countries. A decrease in the relative detection rate of *CDH1* mutations is accepted with a higher absolute number of identified cases [10].

A point not to be neglected within the increased testing, is the “awareness”, e.g., in breast centers; if the lobular subtype is present, a positive family history for breast cancer and a negativity of the standard markers (*BRCA1/2*) is an indication for sequencing *CDH1* and also *CTNNA1* in case of *CDH1* negativity.

### 2.2. Hereditary Lobular Breast Cancer

**Figure 1:**
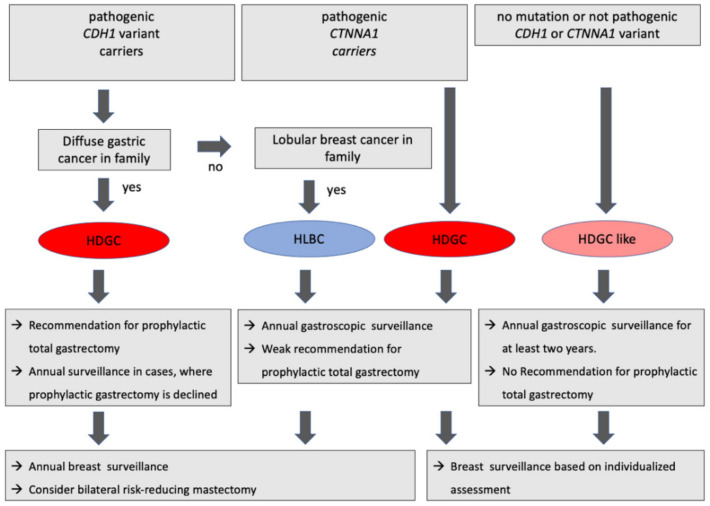
Clinical practice in individuals with positive IGCLC-criteria (HDGC, hereditary diffuse gastric cancer; HLBC, hereditary lobular breast cancer; lGCLC, International Gastric Cancer Linkage Consortium) (according to Blair et al. 2020 [10]).

This group has been newly defined with relevant *CDH1* mutations in individual families, who present only with lobular breast cancers, but not with diffuse gastric cancers. Since the treatment recommendations differ from that of HDGC patients, this entity has been classified as a separate group (Figure 1). Due to the fact that its reclassification within the framework of the IGCLC was performed only recently, little is known on its epidemiology. The *CTNNA1* mutation does not appear to play a role in this group.

### 2.3. Hereditary Diffuse Gastric Cancer-like (HDGC-like)

In around 50% of all cases who fulfilled the family criteria for HDGC (applicable only to family criteria 1 and 2 described in Table 1) *CDH1* or *CTNNA1* mutation was detectable.

For all other cases fulfilling HDGC criteria but without any pathogenic *CDH1* or *CTNNA1* mutation, the HDGC-like group was introduced in 2020. Despite the fact that whole exome sequencing was performed, it cannot be excluded that gene mutations that have been classified as ‘irrelevant’ are causative for this subtype. Separate monitoring criteria have also been introduced in this group (i.e., in contrast to the HDGC group, the precautionary measures are individually adapted to the familial disease patterns (Figure 1)).

## 3. Genetics

### 3.1. CDH1-Mutation

Genetic mutations for the occurrence of HDGC have only been described in the *CDH1* gene and less frequently in the *CTNNA1* gene. In general, genetic testing in childhood or adolescence is not required. Occurrence before 18 years of age has been described only in isolated cases and primarily affects the Maori ethnic group, where screening should be introduced earlier. In principle, screening is recommended in accordance with the respective legal situation (e.g., in Germany starting from the age of 16 at the earliest). The age of the family affected at first manifestation should be considered. The inheritance is autosomal dominant, but the penetrance for the occurrence of gastric cancer is 70% in men and 56% in women [10]. In the case of breast cancer, the *CDH1* analysis should only be performed in the context of the rare lobular subtype, which then entails individualized prevention in the case of positivity (Figure 1). *CTNNA1* gene sequencing needs only to be performed when sequencing of the *CDH1* gene yields no relevant mutations.

### 3.2. CTNNA1-Mutation

There is also little data on the penetrance of diffuse gastric carcinomas and breast carcinomas in *CTNNA1* loss-of-function carriers. Recently, a genetic examination of more than 150,000 individuals revealed that 33 (0.02%) had a *CTNNA1* loss-of-function mutation and of those only 4 (12%) had diffuse gastric cancer, but 22 (67%) had breast cancer [13]. The IGCLC experts therefore recommend annual endoscopic preventive examination and individual breast screening, depending on the family history [10].

## 4. Clinical Approach

### 4.1. HDGC Mutation Carriers without Gastric Cancer

Prophylactic gastrectomy is recommended as standard of care in carriers of a pathogenic *CDH1* mutation and with familial history of proven diffuse gastric cancer. This should be performed in early adulthood up to around 30 years of age. Due to the surgical risk, this recommendation does neither apply to patients over 70 years of age nor with significantly increased peri-operative morbidity. As in the case of HDGC patients, an annual breast check-up or bilateral mastectomy is also recommended. However, in individuals who wish to postpone surgery, annual endoscopic surveillance is accepted as an alternative, searching for subtle alterations such as pale areas, sessile lesions, or erosions. Indeed, there is a lack of prospective data demonstrating a disadvantage of endoscopic surveillance relative to prophylactic gastrectomy [14].

Moreover, little is known on the risk of disease in carriers of a pathogenic CDH1 mutation in the absence of a family history for diffuse gastric cancer but with familial lobular breast carcinoma (HLBC). However, a study on 113 mutation carriers and their 476 relatives showed that this group did not include any cases suffering from gastric carcinomas only, but instead presented with breast and gastric carcinomas in 38% of patients [15]. In this group, prophylactic gastrectomy should therefore be recommended with caution. Here, an annual endoscopy and an annual breast screening or a bilateral mastectomy is recommended. These recommendations also apply to those individuals affected without a positive family history but with evidence of a pathogenic *CDH1* mutation (Figure 1).

In case of positive HDGC criteria and either no (HDGC-like) or uncertain pathogenic evidence for a *CDH1* mutation, the current guideline recommends annual endoscopic monitoring for the duration of two years with a subsequent extension of the follow-up interval and gynecological care that is individually tailored to the family history (Figure 1) [10].

### 4.2. HDGC Mutation Carriers Diagnosed with Gastric Cancer

Patients with evidence of a *CDH1* mutation or patients who meet the IGCLC criteria and who are diagnosed with diffuse gastric cancer should be treated in accordance with the international guidelines [16]. Curative and palliative management do not differ from that of sporadic carcinomas, with the exception of mandatory complete gastrectomy regardless of the location of the tumor in curative intention. In case of curative approach, resection margins should be assessed following the recommendations for prophylactic gastrectomy. HDGC patients treated curatively should receive an annual breast cancer screening or a bilateral mastectomy as part of their follow-up regime.

### 4.3. Screening Endoscopy

The annual screening gastroscopy should ideally be performed in a center with a high level of expertise in HDGC. Examination time of more than 30 min and high-resolution zoom endoscopes are mandatory. For proper assessment of the mucosa, the stomach should first be flushed with mucolytic substances. While the tumor localization in Caucasians is more in the fundus or corpus area, in the Maori ethnic group it is more localized in the antrum. Any suspicious lesion should be biopsied (in particular, mucosa areas appearing pale and those displaying a rigid wall motility during inflation and deflation). According to the recommendations of the IGCLC, at least five random biopsies should be collected from six zones of the stomach each according to the Cambridge biopsy protocol [17]. So far, a single study from the US has provided evidence that the detection rate for signet ring cell foci increases by 36% when the number of random biopsies is further increased to an average of 88 samples per endoscopy [18]. Helicobacter pylori infection should always be treated [19]. Further measures such as laser endomicroscopy, (digital) chromoendoscopy, or endoscopic ultrasound did not show any additional benefit [20,21].

### 4.4. Prophylactic Gastrectomy

After endoscopic exclusion of a carcinoma, the surgical intervention should be carried out as a complete gastrectomy, which implies histological proof for the presence of both the squamous epithelium of the esophagus and the duodenal mucosa at the proximal and distal resection margin, respectively. An extended lymphadenectomy (D2), as it is mandatory morbidity in oncologic gastric surgery, is not recommended, as this contributes to additional. If, however, chances to detect a carcinoma in the specimen are of clinical significance, the authors of the IGCLC guideline pragmatically recommend a D1 lymphadenectomy [11]. Laparoscopic gastrectomy with a pouch is now the standard in gastric cancer surgery [22,23].

## 5. Other Entities

In addition to the classic hereditary gastric cancer with signet ring cells (HDGC), there are regularly patients with intestinal gastric cancer and a positive family history (familiar intestinal gastric cancer (FIGC)) that underlies an autosomal-dominant mode of inheritance. With the exception of an association with the *PALB2* gene, polygenetic tumorigenesis is assumed. Other monogenetic associations have not yet been established, microsatellite instability is found in 38% [4,24]. The screening criteria and the practical procedure are no longer based on the Amsterdam criteria but have been adapted to the recommendations of the IGCLC [8,25]. Genetic mutations that are associated with an increased incidence of gastric cancer were identified in the *STK11* gene (Peutz-Jeghers syndrome), genes connected to microsatellite instability (Lynch syndrome), the p53 tumor suppressor gene (Li-Fraumeni syndrome), and the *APC* gene (GAPSS). The preventive measures are adapted to the respective illness and the family risk.

## 6. Perspective

Current research aims to decipher (i) different penetrance rates, although associated gene mutations appear to be identical; (ii) the mechanisms explaining the progress from a superficial population of signet ring cell foci to invasively growing carcinoma; (iii) identifying optimal intervals and modalities for surveillance endoscopies; and (iv) an awareness campaign aiming for the dissemination of the results of current research to implement concluding recommendations, to educate those affected and to ensure adequate application of screening diagnostics.

## Figures and Tables

**Table 1:** Genetic testing criteria according to the consensus meeting of IGLCL (Blair et al. 2020 [10]).

	**Family Criteria**
1	≥2 cases of gastric cancer in family, independent of age at least >1 proven diffuse subtype ^1^
2	≥1 case of diffuse type gastric cancer any age and ≥1 case of lobular breast cancer diagnosed <70 years of age in different family members ^1^
3	≥2 cases of lobular breast cancer in family (both diagnosed <50 years of age) ^1^
	**Individual Criteria**
1	Individuals with diffuse type gastric cancer aged <50 years.
2	Gastric in situ signet ring cells and/or pagetoid spread of signet ring cells in individuals aged <50 years of age
3	Diffuse type gastric cancer at any age in individuals with a personal or family history (first-degree relative) of cleft lip/cleft palate
4	History of diffuse type gastric cancer and lobular breast cancer, both diagnosed <70 years of age
5	Bilateral lobular breast cancer/lobular carcinoma in situ, both diagnosed <70 years of age
6	Diffuse type gastric cancer at any age in individuals of Maori ethnicity Table 1: Genetic testing criteria according to the consensus meeting of IGLCL (Blair et al. 2020, [10]). ^1^ Family members must be first- or second-degree blood relatives (according to Blair et al. 2020 [10]).

^1^ Family members must be first- or second-degree blood relatives (according to Blair et al. 2020 [10]).
